# Functional and cognitive recovery after intensive care unit-initiated intensive rehabilitation in anti–N-methyl-D-aspartate receptor encephalitis: A long-term case report

**DOI:** 10.1097/MD.0000000000049355

**Published:** 2026-06-19

**Authors:** Yongwun Cho, Hyunggi Park, Young-Soo Kim, Hayoung Byun

**Affiliations:** a Department of Ophthalmology, Gyeongsang National University Hospital and Gyeongsang National University College of Medicine, Jinju, Republic of Korea; b Department of Rehabilitation Medicine, Gyeongsang National University College of Medicine and Gyeongsang National University Hospital, Jinju, Republic of Korea; c Department of Neurology, Gyeongsang National University Hospital, Gyeongsang National University College of Medicine, Jinju, Republic of Korea; d Institute of Medical Science, Gyeongsang National University, Jinju, Republic of Korea.

**Keywords:** autoimmune, case reports, encephalitis, intensive, intensive care unit, rehabilitation

## Abstract

**Rationale:**

Anti-N-methyl-D-aspartate receptor (anti-NMDAR) encephalitis is a severe autoimmune encephalitis that can result in prolonged coma, ventilator dependence, and long-term disability. Although immunotherapy is well established, the optimal timing and intensity of rehabilitation remain poorly defined. This report describes a rare case of adult anti-NMDAR encephalitis with profound neurological impairment who achieved remarkable functional and cognitive recovery following intensive, goal-oriented rehabilitation initiated while still in the intensive care unit (ICU).

**Patient concerns:**

A 35-year-old woman with prior thyroid carcinoma and ovarian teratoma presented with fever, confusion, and behavioral changes progressing to decreased consciousness. She remained comatose for months despite extensive immunotherapy and supportive care.

**Diagnoses:**

Anti-NMDAR encephalitis was confirmed by the detection of anti-NMDAR antibodies in both serum and cerebrospinal fluid. Neuroimaging excluded other causes of encephalopathy.

**Interventions:**

The patient underwent sequential immunotherapy including high-dose corticosteroids, intravenous immunoglobulin, and plasma exchange, followed by rituximab, tocilizumab, and cyclophosphamide. After nearly 20 months of medical instability, she began intensive multidisciplinary rehabilitation—five sessions per week, 2 hours daily—while still in the ICU. Therapy targeted progressive mobilization, upper-limb activation, and communication training, delivered under continuous cardiorespiratory monitoring.

**Outcomes:**

Over 3 months, substantial improvement was observed: Modified Barthel Index increased from 0 to 72, Berg Balance Scale to 42, Mini-Mental State Examination to 9, and Manual Function Test to over 80 bilaterally. She achieved independent ambulation and daily self-care, with significant gains in language function. The patient reported high satisfaction with her regained independence and ability to resume normal activities, and no adverse events occurred during rehabilitation.

**Lessons:**

This case demonstrates that active, structured rehabilitation can be safely implemented even in medically complex, prolonged cases of autoimmune encephalitis. Intensive, goal-directed therapy under ICU monitoring may promote late neuroplasticity and yield meaningful recovery long after disease onset. Rehabilitation should be considered an an integral therapeutic component alongside immunotherapy in the long-term management of severe anti-NMDAR encephalitis.

## 1. Introduction

Anti-N-methyl-D-aspartate receptor (anti-NMDAR) encephalitis is an autoimmune disorder caused by IgG antibodies targeting the NR1 subunit of the N-methyl-D-aspartate (NMDA) receptor, producing a multistage syndrome that progresses from psychiatric and behavioral changes to movement disorders, dysautonomia, and decreased consciousness. Foundational clinical and experimental studies by Dalmau and colleagues established the immunologic mechanism and synaptic receptor internalization underlying this condition.^[1,2]^ Diagnosis is based on the subacute onset of characteristic neuropsychiatric features, supported by cerebrospinal fluid (CSF) and electroencephalographic findings. CSF anti-NR1 IgG remains the most specific biomarker, while the “extreme delta brush” electroencephalogram pattern may assist early recognition.^[3,4]^ Treatment typically follows a stepwise protocol, beginning with high-dose corticosteroids, intravenous immunoglobulin (IVIG), and/or plasma exchange, followed by escalation to rituximab or cyclophosphamide for nonresponders.^[5]^ Interleukin-6 blockade with tocilizumab has been reported as an additional therapeutic option in refractory cases.^[6]^

Despite advances in immunotherapy, many survivors experience persistent cognitive, behavioral, and functional deficits that can last for years.^[7]^ From a rehabilitation perspective, early mobilization in intensive care unit (ICU) settings and multidisciplinary rehabilitation programs have been shown to improve outcomes in severe brain injuries and encephalitis.^[8–10]^ However, detailed adult cases describing long-term functional recovery after prolonged critical illness—particularly those demonstrating delayed but high-intensity rehabilitation—remain exceptionally uncommon, with only a handful of reports published to date. Although most evidence for early rehabilitation comes from stroke and traumatic brain injury, similar principles of early mobilization and task-specific multidisciplinary engagement are increasingly applied to autoimmune encephalitis. Coordinated rehabilitation initiated once patients achieve medical stability, even while under intensive care, may reduce complications and accelerate recovery.

This case is distinguished by the initiation of structured, goal-oriented rehabilitation while the patient remained in the ICU after prolonged mechanical ventilation – a stage typically considered too unstable for active therapy. It provides an opportunity to explore the feasibility, safety, and long-term benefits of initiating rehabilitation under intensive care monitoring and highlights the potential for meaningful functional recovery even after an extended period of severe illness. This study conforms to all CARE guidelines and reports the required information accordingly. This case report was conducted in accordance with the Declaration of Helsinki. Ethical review and approval were waived for this study, as it describes a single clinical case. Informed consent for publication was obtained from the patient.

## 2. Case report

A 35-year-old woman with a history of papillary thyroid carcinoma (treated with total thyroidectomy and radioiodine ablation several years earlier) and a previous right ovarian mature cystic teratoma (surgically treated) was initially well until she developed fever and progressive confusion. Over the following days, she exhibited abnormal behavior, disorientation, and autonomic instability, eventually progressing to decreased consciousness. Anti-NMDAR antibodies were detected in both serum and cerebrospinal fluid, confirming autoimmune encephalitis. There were no significant diagnostic challenges such as limited test access or atypical presentation.

During the acute phase, she received sequential immunotherapy consisting of high-dose corticosteroids, intravenous immunoglobulin, and plasma exchange as first-line treatment. Because of incomplete response, second-line therapy with rituximab, tocilizumab, and cyclophosphamide was administered. Despite these interventions, she remained comatose and dependent on prolonged mechanical ventilation via tracheostomy. Bedside rehabilitation, limited to passive range-of-motion and postural facilitation, began once vital signs stabilized.

Several months later, during her prolonged ICU stay, abdominal imaging revealed a newly developed left adnexal cystic lesion consistent with a mature cystic teratoma, which was subsequently removed. Pathologic findings confirmed a benign teratomatous lesion. Following surgery and gradual neurological stabilization, she continued to receive supportive care in the ICU for an extended period. Formal prognostic staging was not applicable for this disorder; prognosis was inferred from clinical stabilization and functional responsiveness.

Approximately 20 months after disease onset, her condition improved sufficiently to allow transition from supportive bedside management to intensive, goal-oriented, multidisciplinary rehabilitation while she remained in the ICU. The decision was not based on disease duration alone, but on repeated multidisciplinary ICU-rehabilitation assessments of physiologic readiness. At the time of transition, the patient still had a tracheostomy, severe deconditioning, and markedly impaired responsiveness; however, the following safety criteria were satisfied: stable hemodynamics without new or escalating vasoactive support; stable oxygenation and ventilation through a secure tracheostomy without increasing respiratory support; absence of uncontrolled seizures, severe dysautonomia, active infection, bleeding, or unresolved surgical contraindication; secure airway, vascular, and urinary lines; and tolerance of supported sitting or simple active-assisted tasks without cardiorespiratory deterioration. These criteria were consistent with published ICU mobilization safety domains and were used to distinguish preventive bedside rehabilitation from intensive active rehabilitation.^[11,12]^ Rehabilitation sessions were conducted in the rehabilitation treatment room under continuous cardiorespiratory monitoring, with no adverse events observed throughout the program. Thus, ICU-phase implementation of structured rehabilitation demonstrated that active, goal-directed therapy can be feasible in selected medically complex patients when safety criteria are met.

Rehabilitation therapy was provided 5 times per week (about 2 hours per day) and included physical, occupational, and speech-language therapy. Physical therapy emphasized progressive mobilization and upright tolerance using graded support. Occupational therapy focused on upper-limb activation and simple activities of daily living sequencing. Speech-language therapy addressed comprehension and naming tasks using multimodal cues adapted from Western Aphasia Battery hierarchies. All assessments and therapy sessions were supervised by the same multidisciplinary team to ensure consistency and safety. Intensive rehabilitation continued for approximately 8 weeks while the patient remained admitted to the ICU and was maintained after transfer to the general ward, for a total duration of about twelve weeks of structured, goal-oriented therapy. The patient completed all scheduled rehabilitation sessions without interruption, demonstrating good adherence and tolerance.

At the start of intensive rehabilitation, the patient was unable to perform any functional or cognitive task, with Modified Barthel Index, Berg Balance Scale, and mini-mental state examination all effectively 0. Over approximately 3 months of intensive rehabilitation, her Modified Barthel Index improved to 72, Berg Balance Scale to 42, and mini-mental state examination to 9, reflecting significant recovery of postural control, cognitive responsiveness, and independence in daily activities. Hand function, assessed by the Manual Function Test (0–100 scale), improved from 0 to 93.75 on the right and 81.25 on the left, enabling independent self-feeding and personal hygiene with minimal assistance.

By the time of discharge, she achieved independent ambulation on level ground without assistive devices and was able to perform most basic activities of daily living independently. Communication ability, assessed by the Korean version of the Western Aphasia Battery (K-WAB), also improved substantially (Aphasia Quotient 27.6 at baseline), indicating progressive restoration of expressive and receptive language. After recovery, the patient expressed satisfaction with her regained independence and ability to resume normal activities. Although residual fine motor and higher-order cognitive deficits persisted, her overall trajectory represented a transition from total dependence to near-functional independence. These findings highlight the long-term rehabilitative potential achievable even after a protracted and medically complex disease course.

## 3. Discussion

This case highlights that meaningful recovery is possible in severe, chronic anti-NMDAR encephalitis when intensive, goal-oriented rehabilitation is implemented after medical stabilization, even if the patient remains in the ICU setting. Unlike previously reported cases, this patient underwent intensive, goal-directed rehabilitation while still in the ICU after nearly 2 years of medical instability, representing an unusually long interval between disease onset and meaningful functional recovery in adult anti-NMDAR encephalitis. Previous reports have underscored the importance of multidisciplinary rehabilitation; however, few have described recovery following delayed yet high-intensity intervention after prolonged critical illness.^[8–10]^ The present case demonstrates that, even after an extended period of medical instability, structured rehabilitation can produce measurable and durable functional gains when delivered under coordinated ICU-phase management.

The timing of rehabilitation initiation in severe anti-NMDAR encephalitis should be staged rather than defined solely by ward transfer or normalization of consciousness. Preventive bedside rehabilitation, including positioning, passive range-of-motion exercise, postural facilitation, and pressure-injury prevention, is indicated once basic vital-sign stability is achieved. Intensive active rehabilitation should be considered when the patient demonstrates stable cardiorespiratory status, controlled dysautonomia and seizures, absence of active systemic or surgical contraindications, secure airway and lines, and tolerance of graded mobilization under ICU monitoring. These criteria correspond to ICU mobilization safety domains encompassing respiratory, cardiovascular, neurologic, and other patient-specific risks.^[11,12]^ The indication for intensive rehabilitation in such severe cases is therefore the combination of prolonged immobilization, emerging physiologic tolerance to activity, potentially reversible neurologic disease, and acceptable risk under close multidisciplinary monitoring. Early physical and occupational therapy in mechanically ventilated ICU patients has been associated with improved functional outcomes, supporting the principle that rehabilitation should be integrated into critical care when safety thresholds are satisfied.^[13]^

The timing of therapeutic intervention should also be interpreted at 2 levels in this case: immunologic treatment and active rehabilitation. Previous anti-NMDAR encephalitis cohorts have shown that early immunotherapy and tumor removal, when applicable, are associated with better long-term outcomes.^[5]^ In adult patients requiring intensive care, early immunotherapy after ICU admission has been associated with favorable neurologic outcomes, and prognostic models such as the NEOS score include treatment delay and ICU admission among predictors of poor 1-year functional status.^[14,15]^ In our patient, sequential immunotherapy was administered during the acute disease course and escalated because of an incomplete response; however, intensive active rehabilitation was delayed until approximately 20 months after onset because persistent impaired consciousness, tracheostomy-dependent respiratory care, dysautonomia, systemic complications, and surgical issues precluded consistent active participation. This delay likely contributed to profound deconditioning, ICU-acquired weakness, cognitive-linguistic understimulation, and total dependence at the start of rehabilitation. Had medical stability permitted earlier initiation of active rehabilitation, the rate and extent of recovery might have been greater by limiting secondary deconditioning and cognitive-linguistic understimulation; nonetheless, the observed improvement suggests that delayed intensive rehabilitation may still harness residual neuroplastic capacity and support meaningful functional recovery. Therefore, the present case does not support delaying rehabilitation when safe mobilization is possible. Instead, it suggests that meaningful gains may still occur after delayed stabilization if repetitive, goal-directed, multidisciplinary therapy is implemented.

Substantial recovery after prolonged consciousness disturbance and immobilization is uncommon but biologically plausible in selected patients with anti-NMDAR encephalitis. A recent international cohort of patients with anti-NMDAR encephalitis who remained in a vegetative state for at least 9 months reported median durations of vegetative state, ICU stay, and mechanical ventilation of 399, 275, and 270 days, respectively; after extended follow-up, full or substantial recovery was achieved in approximately 2-thirds of cases.^[16]^ This disease-specific evidence suggests that prolonged impaired consciousness does not necessarily indicate irreversible neurologic injury in anti-NMDAR encephalitis. Nevertheless, recovery is not universal, and age, disease severity, complications, treatment responsiveness, and rehabilitation access remain important prognostic considerations. The present case should therefore be interpreted as evidence of feasibility and potential, not as a guarantee of recovery after prolonged coma.

Several mechanisms may explain the late recovery observed in the present case. Anti-NMDAR antibodies can cause a selective and potentially reversible reduction in surface and synaptic NMDAR density rather than fixed neuronal destruction, providing a biological basis for delayed improvement after immunologic control.^[17]^ Functional and structural network alterations reported in anti-NMDAR encephalitis may provide a network-level substrate for persistent deficits and subsequent recovery.^[18]^ Intensive rehabilitation may have contributed by providing repeated, goal-directed sensorimotor, cognitive, and language stimulation, thereby promoting experience-dependent plasticity, strengthening spared networks, and reversing secondary complications of prolonged immobilization.^[19]^ Similar outcomes have been reported in limited pediatric and adult anti-NMDAR encephalitis rehabilitation reports, where intensive physical, cognitive, and language rehabilitation facilitated progressive recovery despite protracted disease duration.^[10,20]^ These mechanisms remain hypothetical in this single case because serial neuroimaging, electrophysiologic measures, and biomarker correlations were not available. However, the close temporal association between the transition from supportive bedside care to structured intensive rehabilitation and the patient’s functional gains supports a clinically meaningful rehabilitative contribution.

From a systems perspective, implementing rehabilitation within the ICU requires structural and cultural collaboration between departments. In this case, the close partnership between rehabilitation and critical care teams enabled safe delivery of therapy under hemodynamic and respiratory monitoring, illustrating that such programs can be feasible in tertiary centers. As the population of autoimmune encephalitis survivors grows, establishing standardized ICU-phase rehabilitation protocols may help reduce long-term disability and healthcare burden.

Limitations include the single-case design, absence of serial neuroimaging, lack of electrophysiologic or biomarker correlates of recovery, and the inability to fully separate the effects of rehabilitation from delayed immunotherapy effects or spontaneous neural repair. Because spontaneous delayed recovery cannot be excluded, the observed improvement should not be attributed solely to rehabilitation. Nonetheless, the detailed functional trajectory documented in this case suggests that targeted rehabilitation may amplify recovery during a still-open neuroplastic window even after a protracted disease course. Future research should focus on defining objective biomarkers of recovery, establishing safe thresholds for ICU-based rehabilitation in autoimmune encephalitis, and evaluating long-term outcomes in larger cohorts.

## 4. Conclusions

In conclusion, this case demonstrates that intensive, goal-oriented rehabilitation can contribute to meaningful recovery even after delayed rehabilitation initiation and prolonged medical instability in severe anti-NMDAR encephalitis. Although earlier rehabilitation should be pursued whenever safety criteria are met, prolonged delay should not automatically be interpreted as therapeutic futility. Despite nearly 2 years of critical illness, the patient achieved substantial motor, cognitive, and communicative improvement, reflecting the persistence of neuroplastic potential in selected patients. Rehabilitation should not be regarded merely as a post-acute adjunct but as an integral therapeutic component that is planned alongside immunotherapy and initiated progressively according to ICU safety criteria. Early collaboration between rehabilitation and critical care teams may ultimately improve the long-term standard of care for this complex and often devastating condition.

## Author contributions

**Conceptualization:** Young-Soo Kim, Hayoung Byun.

**Data curation:** Hyunggi Park, Hayoung Byun.

**Formal analysis:** Hayoung Byun.

**Investigation:** Hayoung Byun.

**Methodology:** Young-Soo Kim.

**Supervision:** Hayoung Byun.

**Validation:** Young-Soo Kim.

**Writing – original draft:** Yongwun Cho, Hayoung Byun.

**Writing – review & editing:** Yongwun Cho, Hyunggi Park, Young-Soo Kim, Hayoung Byun.
